# Exploring the impact of a KCNH2 missense variant on Long QT syndrome: insights into a novel gender-selective, incomplete penetrance inheritance mode

**DOI:** 10.3389/fgene.2024.1409459

**Published:** 2024-05-30

**Authors:** Peng Chen, Zainul Zampawala, Hong Wang, Luyun Wang

**Affiliations:** ^1^ Division of Cardiology, Departments of Internal Medicine and Genetic Diagnosis Center, Tongji Hospital, Tongji Medical College, Huazhong University of Science and Technology, Wuhan, China; ^2^ Hubei Key Laboratory of Genetics and Molecular Mechanism of Cardiological Disorders, Wuhan, China

**Keywords:** kcnh2, Long QT syndrome, gender selective, turner syndrome, incomplete penetrance

## Abstract

**Background:**

Long QT syndrome (LQTS) is an inherited malignant arrhythmia syndrome that poses a risk of sudden death. Variants in the Potassium Voltage-Gated Channel Subfamily H Member 2 (*KCNH2*) gene are known to cause Long QT syndrome through an autosomal dominant inheritance pattern. However, as of now, there have been no reports of any *KCNH2* variant leading to Long QT syndrome exhibiting incomplete penetrance that is influenced by gender.

**Methods:**

Whole-exome sequencing (WES) was conducted on the proband to identify pathogenic variants. Subsequently, Sanger sequencing was employed to validate the identified likely pathogenic variants in all family members.

**Results:**

We analyzed a pedigree spanning three-generations afflicted by Long QT syndrome. WES revealed a novel *KCNH2* missense variant (p.Val630Gly, c.1889 T>G) as the causative factor for the family’s phenotype. Within this family, all three male carriers of the *KCNH2* variant carriers exhibited the Long QT syndrome phenotype: one experienced sudden death during sleep, another received an implantable cardioverter defibrillator (ICD), and a younger man displayed a prolonged QTc interval without any instances of syncope or malignant arrhythmia to date. Interestingly, the middle-aged female carrier showed no Long QT Syndrome phenotype. However, her offspring, diagnosed with Turner syndrome (45, X) and also a carrier of this variant, experienced frequent syncope starting at 12 years old and was diagnosed with Long QT syndrome, leading to an ICD implantation when she was 15 years old. These observations suggest that the manifestation of Long QT syndrome associated with this KCNH2 variant exhibits incomplete penetrance influenced by gender within this family, indicating potential protective mechanisms against the syndrome in females affected by this variant.

**Conclusion:**

Our investigation has led to the identification of a novel pathogenic *KCNH2* variant responsible for Long QT syndrome within a familial context characterized by gender-selective, incomplete penetrance. This discovery highlights a unique pathogenic inheritance pattern for the *KCNH2* gene associated with Long QT syndrome, and could potentially shed light on the distinct penetrance behaviors and patterns of the *KCNH2* gene. This discovery broadens our exploration of the KCNH2 gene in cardiac arrhythmias, highlighting the intricate genetic dynamics behind Long QT syndrome.

## Introduction

Long QT syndrome (LQTS) is an inherited arrhythmia syndrome, which characterized by a prolonged corrected QT interval on the Electrocardiogram (ECG) without structural heart disease and drug influence (Priori et al., 2013; Schwartz and Ackerman, 2013). LQTS is associated with malignant arrhythmias that can lead to syncope and sudden death (Schwartz, 2021). Most patients with LQTS have pathogenic variants in genes encoding ion channels or their regulating proteins (Wilde et al., 2022). The three most common subtypes of LQTS are distinguished based on characteristic features and genotype-phenotype relationships, which have been widely described (Tester et al., 2005; Kapplinger et al., 2009).

LQT2, accounting for approximately 30% of LQTS cases, exhibits high penetrance (Wilde et al., 2022). Loss-of-function variants in potassium voltage-gated channel subfamily H member 2 (*KCNH2*) gene are responsible for LQTS2 (Wilde et al., 2022). To date, a significant number of pathogenic or likely pathogenic *KCNH2* variants have been reported, with the majority showing no gender-specific differences in morbidity. However, select studies have reported that females may possess a heightened risk of developing morbidity and life-threatening arrhythmias associated with LQTS2 (Mazzanti et al., 2018; Ke et al., 2023).

Turner syndrome is a rare genetic disorder characterized by features such as female hypergonadotropic hypogonadism, dysplasia, infertility, endocrine and a spectrum of endocrine and metabolic syndromes (Gravholt et al., 2019). This condition arises from the complete or partial absence of one X-chromosome, leading to the pathogenesis of Turner syndrome (Barr and Bertram, 1949). Consequently, individuals with Turner syndrome may exhibit features typically associated with “maleness” (Steiner and Saenger, 2022). Additionally, there is an increased risk of cardiovascular diseases in these patients, including hypertension, coronary heart disease, heart failure, and aortic disease (Mortensen et al., 2018).

In this study, we identified a novel pathogenic *KCNH2* variant through whole exome sequencing in a three-generation pedigree. This variant causes LQTS with a novel-gender selective, incomplete penetrance inheritance pattern.

## Methods

### Participant recruitment

We identified a three-generation pedigree with multiple individuals suffering from LQTS. This family was recruited at Tongji Hospital in 2021, with the Institutional Review Board of Tongji Hospital granting approval for the study. Additionally, we recruited 500 healthy, unrelated Han Chinese individuals’ as controls from those undergoing routine health examinations. Informed consent, including consent for portraits, was obtained from all participants. ECGs were collected from all family members. Echocardiography and 24-h Holter monitoring were specifically performed for the proband. Furthermore, program-controlled telemetry for the implantable cardioverter defibrillator (ICD) of the proband was conducted regularly. The QT intervals were measured using the Tangent method, and the Bazett correction formula was applied to calculate the rate-corrected QT (QTc) (Vink et al., 2018). The diagnosis of LQTS was determined based on the Schwartz scoring scale (Priori et al., 2015; Westphal et al., 2020).

### Whole exome sequencing

Genomic DNA was extracted from the leukocytes of peripheral blood samples using the QIAamp DNA Mini Kit (Qiagen, Germany). Following extraction, the DNA underwent fragmentation through sonification. The fragmented DNA’s tail ends were subsequently repaired, and adapters were ligated using the Agilent SureSelect Human All Exon V6 Kit (Agilent, Santa Clara, CA). These prepared libraries were then amplified and sequenced on the Illumina HiSeq platform (Illumina, California, America).

### Bioinformatics analysis

The sequence reads were mapped to the GRCh37 human reference genome using the Burrows–Wheeler Aligner (BWA) (Li and Durbin, 2010). Coverage analysis was performed using BWA, while the Genome Analysis Toolkit (GATK) (McKenna et al., 2010) was used for marking duplicates, sorting BAM files, recalibrating base quality scores, and variant calling. Variants were then annotated using ANNOVAR and reported following the Human Genome Variation Society nomenclature guidelines (http://www.hgvs.org/mutnomen). Their evaluation was based on the American College of Medical Genetics (ACMG) standards (Richards et al., 2015). Given that LQTS is a rare inherited condition, variants displaying minor allele frequencies greater than 1% were excluded, as they were deemed unlikely to be deleterious. Population allele frequency data for variants were obtained from databases such as the 1000 Genomes Project (http://browser.1000genomes.org/), the Exome Aggregation Consortium (ExAC) (http://exac.broadinstitute.org/), the Exome Sequencing Project (ESP) (https://evs.gs.washington.edu/EVS/), and the Genome Aggregation Database (gnomAD) (https://gnomad.broadinstitute.org/). Variants also underwent filtering against our exome database of 500 healthy Han Chinese controls to further refine the analysis. The pathogenicity of all variants was assessed through databases like the Human Gene Mutation Database (HGMD) (http://www.hgmd.cf.ac.uk/ac/index.php) and ClinVar database (http://www.ncbi.nlm.nih.gov/clinvar). Additionally, all missense variants were evaluated based on their effect on the function of the coding protein using SIFT, PolyPhen2 HDIV, PolyPhen2 HVAR, LRT, MutationTaster, MutationAssessor, FATHMM, PROVEAN, MetaSVM, MetaLR, VEST, M-CAP, CADD, GERP++, DANN, fathmm-MKL, Eigen, GenoCanyon, fitCons, PhyloP and SiPhy scores. (Chen et al., 2020). Protein structure of mutation was predicted using Pymol. Briefly, the pdb file (A0PJW5) of KCNH2 was downloaded from Alphafold Protein Structure Database (https://alphafold.ebi.ac.uk/) and mutagenesis as well as plot was generated with Pymol.

### Sanger sequencing validation

Sanger sequencing was conducted using Applied Biosystems 3500xl capillary sequencer (Applied Biosystems, Foster City, USA) to validate all variants identified as likely pathogenic and associated with the phenotypes observed in this pedigree. Furthermore, each variant confirmed as likely pathogenic was subjected to Sanger sequencing in a group of 500 healthy controls for further validation.

### Statistical analysis

Statistical analysis was performed using the SPSS software, version 26.0. Group differences were assessed with the Pearson chi-square test, and a *p*-value <0.05 was considered statistically significant.

## Results

### Case presentation

**FIGURE 1:**
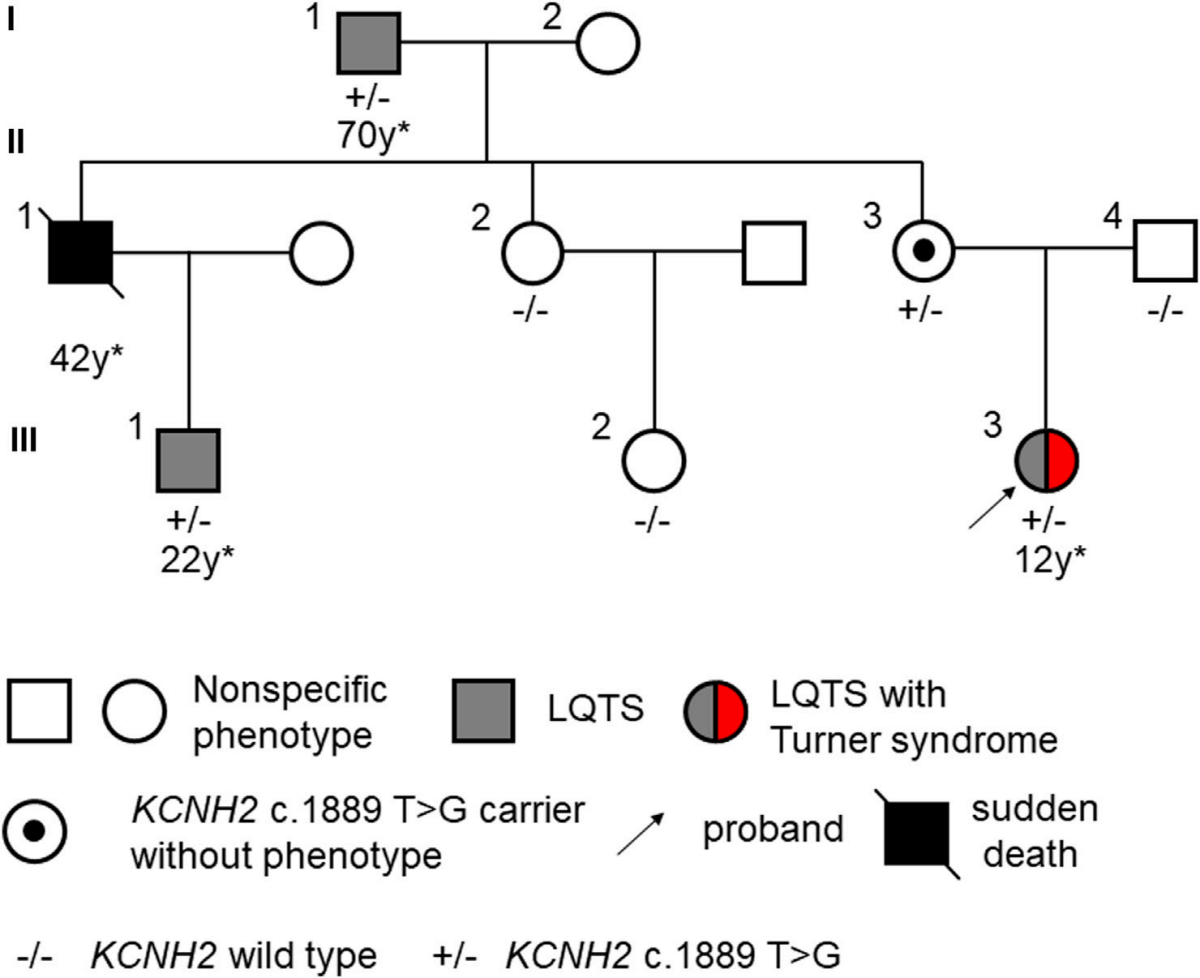
Pedigree of LQTS displaying gender-selective incomplete penetrance. The asterix (*) indicates the age at the first onset of a malignant event or diagnosis of LQTS.

**FIGURE 2:**
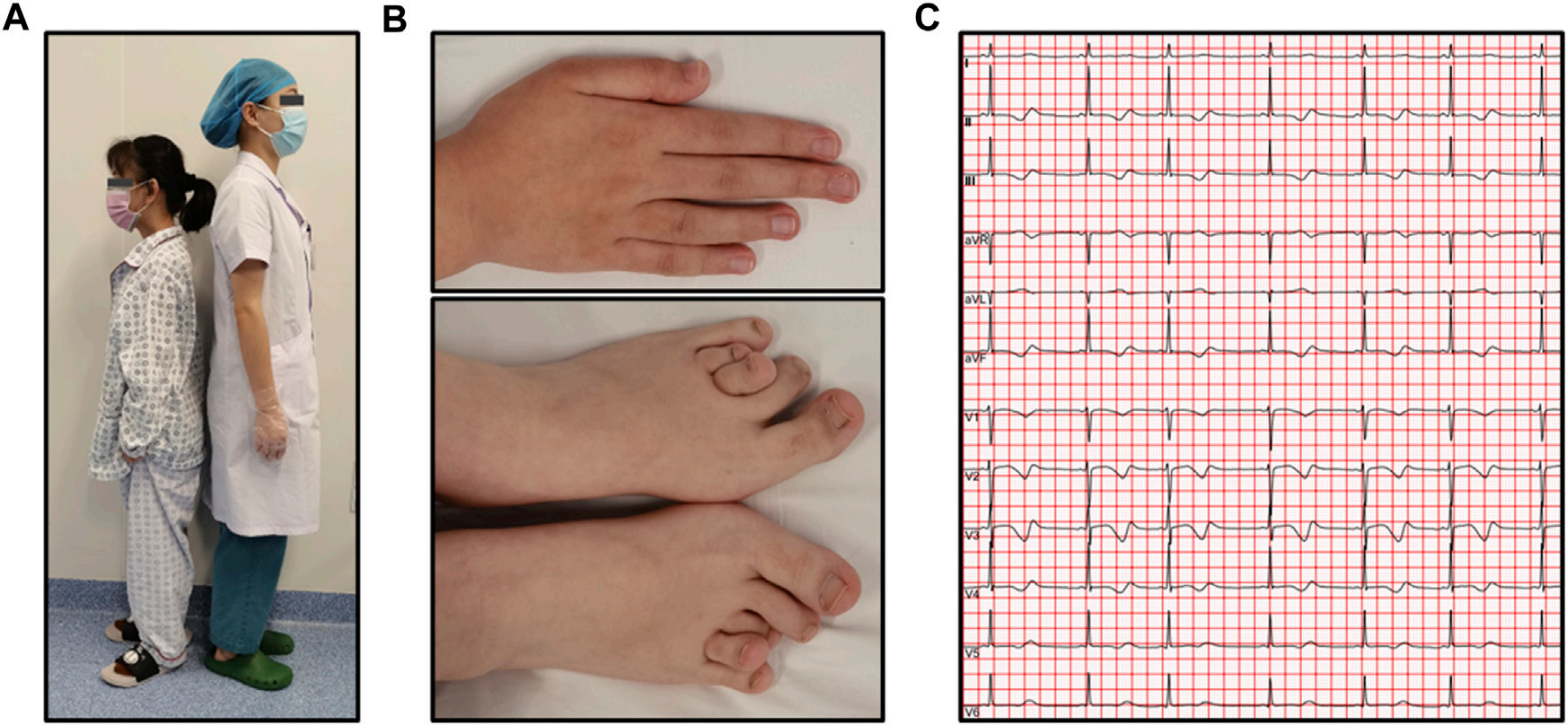
Phenotypes of the proband affected by Turner syndrome. **(A)** Short stature compared to a reference female model, whose height is 160 cm on the right side of the panel; **(B)** Skeletal dysplasia caused by Turner syndrome; **(C)** An ECG demonstrating a prolonged QTc interval with T-wave alterations in multiple leads.

In our study, we identified a three-generation Chinese family presenting multiple cases of LQTS exhibiting gender-selective incomplete penetrance (Figure 1). The proband (III-3), a 15-year-old female with short stature and skeletal dysplasia, has a medical history of Turner syndrome and diabetes (Figures 2A,B). She has been on regular estrogen and metformin therapy. The proband reported symptoms of palpitations and amaurosis over 3 years, accompanied by four episodes of syncope—two occurring during the day with cold sweats and two at night, accompanied by urinary incontinence. Upon admission, a 12-lead electrocardiogram (ECG) revealed a prolonged QTc interval of 485 m for the proband. Further, a 24-h Holter monitor recorded a maximum QTc interval of 518 m, with alterations in the T wave observed across multiple leads (Figure 2C). Echocardiography examination showed no abnormalities, with a left ventricular dimension of 40 mm and an ejection fraction of 61%. Therefore, the diagnosis of this patient was considered as LQTS.

The proband’s grandfather (I-1) began to experience syncope at the age of 70 and received an implantable cardioverter defibrillator (ICD) 2 years later. The proband’s uncle (II-1) suffered multiple syncope episodes starting at the age of 42, but did not undergo any specific treatment. Tragically, he passed away in his sleep that same year. Furthermore, this man’s son (III-1), who had never shown symptoms of palpitation, amaurosis, or syncope, was discovered to have a prolonged QTc interval of 498 m with notched T waves at the age of 22 (Supplementary Figure S1). Meanwhile, the proband’s grandmother (I-2), aunt (II-2), mother (II-3), father (II-4) and cousin sister (III-2) showed no specific symptoms, displaying normal QTc intervals in their 12-lead ECGs. No history of specific cardiology diseases was found in the paternal lineage of the proband. The clinical characteristics of the family members are listed in Table 1.

**TABLE 1:** Clinical characteristics of all members of this pedigree.

Family member	Sex	Age (y)	QT/QTc (ms)	Clinical symptoms	Schwartz-score	Genotype
I-1	M	70^a^	450/472	syncope, malignant arrhythmia, ICD implanted	6	*KCHN2* c.1889 T>G
I-2	F	70	342/417	no	1	WT
II-1	M	42^a^	NA	syncope, SCD	2	NA
II-2	F	47	338/409	no	1	WT
II-3	F	45	408/429	no	1	*KCHN2* c.1889 T>G
II-4	F	46	346/442	no	1	WT
III-1	M	22^a^	424/498	no (notched T waves)	5	*KCHN2* c.1889 T>G
III-2	F	19	388/407	no	1	WT
III-3	F	12^a^	452/485	syncope, malignant arrhythmia, ICD implanted	7	*KCHN2* c.1889 T>G
						Turner syndrome (45, X)

^a^ Indicates the age at the first onset of malignant event or diagnosis of LQTS; F, female; ICD, implantable cardioverter defibrillator; M, male; NA, not available; SCD, sudden cardiac death; WT, wild type.

### Genetic screening

**FIGURE 3:**
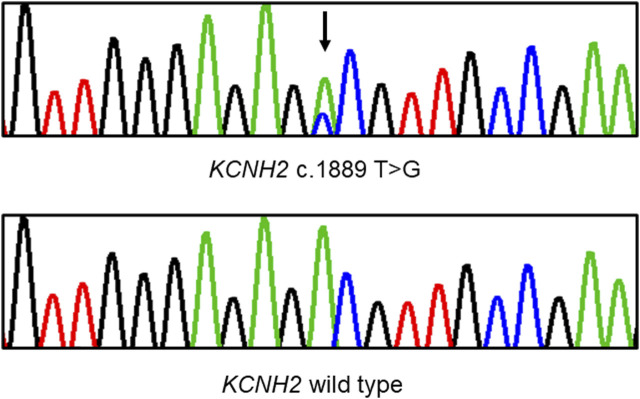
Sanger sequencing demonstrates a heterozygous missense variant in the *KCNH2* gene within the pedigree. A black arrow indicates the position of the mutation.

**FIGURE 4:**
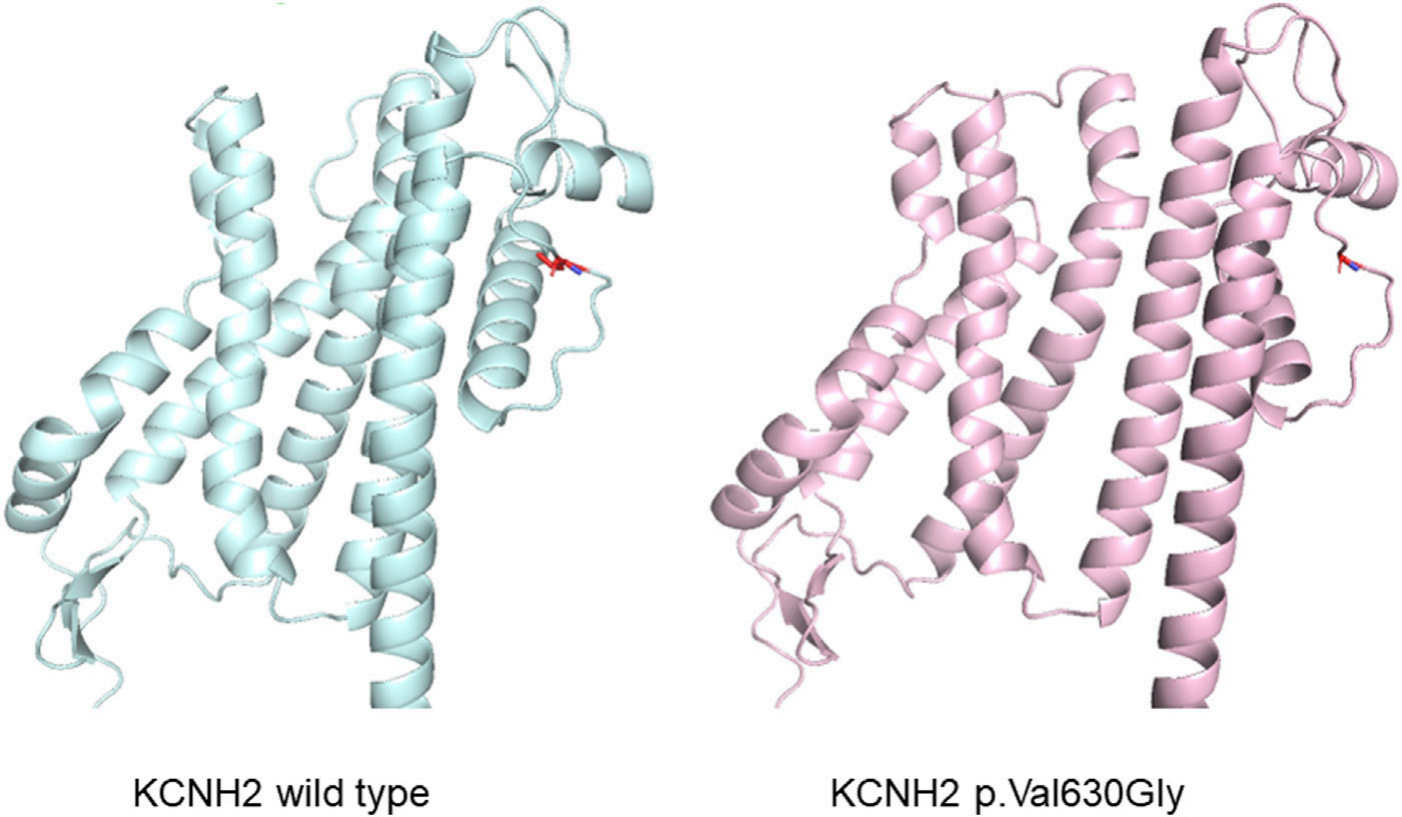
Protein structure prediction illustrating an alteration in the structure of the *KCNH2* protein caused by the variant.

Whole exome sequencing was conducted on the proband, revealing a chromosomal pattern of 45, X, which is consistent with the diagnosis of Turner syndrome. Moreover, a novel missense variant in the *KCNH2* gene (p.Val630Gly, c.1889 T>G) was identified and deemed responsible for the LQTS phenotype observed in this family (Figure 3). This variant has not previously been reported in the 1000 Genomes Project, ExAC, gnomAD, ESP, HGMD, or ClinVar databases, nor was it found in the 500 healthy controls. The missense constraint metrics of *KCNH2* from gnomAD database (missense Z score = 2.48) is quite high, indicating that *KCNH2* is intolerance to missense variants. Additionally, this variant is predicted to be deleterious by *in silico* algorithm (Supplementary Table S1). Protein structure predictions suggest that this variant might alter the structure of KCNH2 protein (Figure 4). Sanger sequencing confirmed the presence of this variant in all relevant family members. The proband’s grandfather (I-1), uncle (II-1), mother (II-3), and cousin brother (III-1) were identified as carriers of the *KCNH2* variant. In contrast, the genotype of the proband’s grandmother (I-2), aunt (II-2), father (II-4) and cousin sister (III-2) were validated as wild type (Table 1; Figure 3). Additionally, whole exome sequencing performed on the proband’s grandfather (I-1), mother (II-3), and cousin brother (III-1) revealed no other pathogenic variants in *KCHN2* or any other genes known to be associated with LQTS. The *KCNH2* gene is recognized as a known causative gene of LQTS. The identified variant co-segregated with the LQTS phenotype in the proband with Turner syndrome and all male family members, except for the proband’s mother. Interestingly, no LQTS phenotypes were observed in the only ‘confirmed’ female carrier of this variant. These findings suggest the existence of protective mechanisms that shields females from LQTS associated with this variant, indicating a gender-selective incomplete penetrance in this pedigree.

### Treatment and follow-up

The ICD was implanted in the proband at the age of 15. Additionally, 10 mg propranolol has been taken for three times daily. In the seventh month after ICD implantation, 52 occurrences of ventricular flutter and 40 episodes of ventricular fibrillation were documented, resulting in five defibrillation events. She was admitted to hospital and diagnosed as electrolyte disturbance (hypokalemia) caused by diarrhea. She was treated with potassium chloride solution, suitable fluid supplement. The ventricular arrythmia attenuated and she recovered promptly. She kept on regular taking propranolol to control the ventricular arrythmia. After that, several occurrences of ventricular flutter, but no defibrillation events again in the first-year follow-up.

## Discussion

This study discovered a novel missense variant in *KCNH2* gene (p.Val630Gly, c.1889T>G) through whole exome sequencing in a three-generation pedigree affected by LQTS. Among the family members carrying this variant, all male carriers and one proband with Turner syndrome exhibited LQTS phenotypes, while the female carrier displayed no abnormalities. Therefore, a novel gender-selective, incomplete penetrance inheritance mode was observed in this pedigree.

The *KCNH2* gene located on chromosome seven which encodes the α-subunit of the voltage-gated K^+^ channel Kv11.1, which comprises six α-helical transmembrane segments (S1-S6). The segments from S1 to S4 constitute the voltage sensor domain, and S5 to S6 form the pore domain (Sanguinetti et al., 1995; Trudeau et al., 1995; Wang and MacKinnon, 2017). This gene is responsible for the rapid delayed rectifier current I_Kr_ (Wilde et al., 2022). Loss-of-function variants in *KCNH2* can decrease the amplitude of I_Kr_, leading to a prolonged ventricular action potential duration (Ono et al., 2020). Previous studies have shown that more than 90% of variants in *KCNH2* disrupt the intracellular transport or trafficking of the Kv11.1 channel to the cell surface membrane (Ono et al., 2020). In our study, referencing the SMART database (http://smart.embl-heidelberg.de/), the identified *KCNH2* variant (p.Val630Gly, c.1889 T>G) is associated with a transmembrane region in S5-S6, potentially affecting the spatial structure and functionality of the pore domain.

Previous studies have highlighted gender differences in the manifestation of LQTS. Notably, a comprehensive follow-up study including 1,710 LQTS patients indicated that females are at a higher risk of experiencing life-threatening arrhythmic events (Mazzanti et al., 2018). Additionally, a case report outlined a pedigree with a putative *KCNH2* mutation that exclusively affected females (Ke et al., 2023). Our research similarly identified a family with LQTS carrying a *KCNH2* variant, which exhibited gender-selective incomplete penetrance. Contrary to expectations, female carriers of the *KCNH2* variant did not exhibit LQTS phenotypes; instead, male carriers and those with Turner syndrome (45, X) demonstrated typical LQTS phenotypes. Previous studies suggested that this gender-selective pattern might be influenced by androgen levels, considering the differential impact of sex hormones on potassium currents (Grouthier et al., 2021; Ke et al., 2023). However, our findings challenge this theory, as the Turner syndrome patient, despite undergoing regular estrogen therapy for years, began experiencing arrhythmias at a younger age compared to all male patients in the study. This observation leads us to speculate that the presence of complete female sex chromosomes (XX) could offer protective mechanisms against the development of LQTS in carriers of this variant. Supporting this, published data indicate that male LQTS2 patients with mutations in the pore region are at a higher risk of cardiac event than those with non-pore mutations (Platonov et al., 2018). This observation may partially explain the gender-selective incomplete penetrance inheritance pattern observed in this family’s pedigree.

A previous study demonstrated that most LQTS2 patients start showing symptoms around puberty (Wilde et al., 2022). In this family we studied, male patients began experiencing arrhythmias at an older age. However, we observed a trend where the onset of malignant arrhythmias or sudden cardiac death occurred progressively earlier with each generation. Despite this, the proband (III-3), who has Turner syndrome, started suffering from malignant arrhythmias at a significantly younger age than other family members, displaying symptoms more frequently. Prior studies have shown that Turner syndrome patients are at a higher risk of cardiovascular diseases, including hypertension, congenital heart disease, coronary heart disease, and aortic disease (De Groote et al., 2017; Klaskova et al., 2017; Mortensen et al., 2018). This suggests that Turner syndrome may cause latent cardiac structural changes, potentially hastening the onset of LQTS in the proband or affected individuals.

### Limitation

This study identified a LQTS pedigree exhibiting a gender-selective incomplete penetrance inheritance pattern. However, a limitation of our study is the absence of a second female carrier of the variant within this pedigree. Future research should include functional studies on this variant to deepen our understanding.

## Conclusion

This study identified a novel missense variant in the *KCNH2* gene, linked to LQTS in a Chinese Han family/pedigree, characterized by gender-selective incomplete penetrance. This suggests the existence of protective mechanisms that may shield female carriers from the onset of LQTS. The discovery of this variant introduces a novel pathogenic inheritance mode for *KCNH2* associated LQTS, offering valuable insights into the gene’s penetrance mechanisms. This contribution enriches our understanding of the genetic factors influencing LQTS and highlights the importance of considering gender in genetic studies of inherited arrhythmias.

## Data Availability

The data presented in the study are deposited in the following depository: https://pan.baidu.com/s/1-SEpn19b49lPxVfkZr5uyw acess code: 5dpt
