# Supplementary material for: Exploring the impact of a KCNH2 missense variant on Long QT syndrome: insights into a novel gender-selective, incomplete penetrance inheritance mode
Source: Front Genet. 2024 May 30;15:1409459. doi: 10.3389/fgene.2024.1409459 (PMC11169575; doi:10.3389/fgene.2024.1409459)
Supplement: Supplementary file 1 [file DataSheet1.docx]

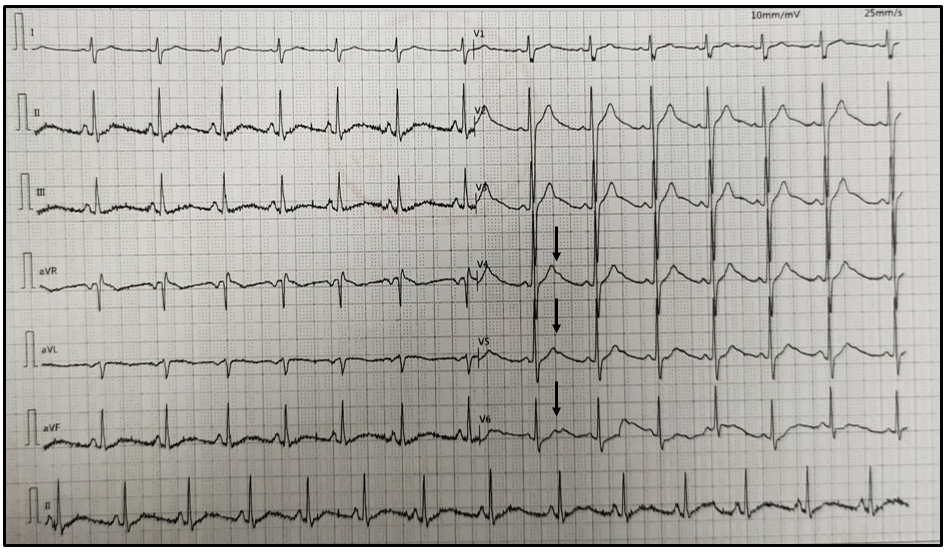


**Supplementary Figure 1.** A 12-lead ECG of individual III-1 displaying a prolonged QTc interval with notched T waves. Arrows highlight the locations of the notched T waves.

**Supplementary Table 1.** The prediction of the *KCNH2* variant by ANNOVAR.

| **Position** | **Amino acids change** | | **SIFT** | **PolyPhen2 HDIV** | **PolyPhen2 HVAR** | **LRT** | **MutationTaster** | **MutationAssessor** | **FATHMM** |
| --- | --- | --- | --- | --- | --- | --- | --- | --- | --- |
| chr7:150648592 | *KCNH2*:NM_000238:exon7:c.1889T>G:p.V630G  *KCNH2*:NM_001204798:exon3:c.869T>G:p.V290G  *KCNH2*:NM_172057:exon3:c.869T>G:p.V290G  *KCNH2*:NM_172056:exon7:c.1889T>G:p.V630G | | Damaging | Probably damaging | Probably damaging | Deleterious | Disease causing | High | Damaging |
| **FathmmMKL coding** | **PROVEAN** | **MetaSVM** | **MetaLR** | **M-CAP** | **REVEL** | **PrimateAI** | **ClinPred** | **MVP** | **DEOGEN2** |
| Deleterious | Deleterious | Damaging | Damaging | Damaging | Damaging | Damaging | Damaging | Damaging | Damaging |
| **LIST-S2** | **VEST4** | **CADD** | **DANN** | **Eigen** | **Eigen-PC** | **GERP++** | **phyloP** | **phastCons** | **SiPhy** |
| Damaging | Damaging | Damaging | Damaging | Damaging | Damaging | Conserved | Conserved | Conserved | non-Conserved |
